# Gadolinium-enhanced cardiac MR exams of human subjects are associated with significant increases in the DNA repair marker 53BP1, but not the damage marker γH2AX

**DOI:** 10.1371/journal.pone.0190890

**Published:** 2018-01-08

**Authors:** Jennifer S. McDonald, Robert J. McDonald, Jacob B. Ekins, Anthony S. Tin, Sylvain Costes, Tamara M. Hudson, Dana J. Schroeder, Kevin Kallmes, Scott H. Kaufmann, Philip M. Young, Aiming Lu, Ramanathan Kadirvel, David F. Kallmes

**Affiliations:** 1 Department of Radiology, College of Medicine, Mayo Clinic, Rochester, MN, United States of America; 2 Exogen Biotechnology Inc., Berkeley, CA, United States of America; 3 Department of Molecular Pharmacology and Experimental Therapeutics, College of Medicine, Mayo Clinic, Rochester, MN, United States of America; 4 Department of Oncology, College of Medicine, Mayo Clinic, Rochester, MN, United States of America; 5 Department of Neuroscience, College of Medicine, Mayo Clinic, Rochester, MN, United States of America; CNR, ITALY

## Abstract

Magnetic resonance imaging is considered low risk, yet recent studies have raised a concern of potential damage to DNA in peripheral blood leukocytes. This prospective Institutional Review Board-approved study examined potential double-strand DNA damage by analyzing changes in the DNA damage and repair markers γH2AX and 53BP1 in patients who underwent a 1.5 T gadolinium-enhanced cardiac magnetic resonance (MR) exam. Sixty patients were enrolled (median age 55 years, 39 males). Patients with history of malignancy or who were receiving chemotherapy, radiation therapy, or steroids were excluded. MR sequence data were recorded and blood samples obtained immediately before and after MR exposure. An automated immunofluorescence assay quantified γH2AX or 53BP1 foci number in isolated peripheral blood mononuclear cells. Changes in foci number were analyzed using the Wilcoxon signed-rank test. Clinical and MR procedural characteristics were compared between patients who had a >10% increase in γH2AX or 53BP1 foci numbers and patients who did not. The number of γH2AX foci did not significantly change following cardiac MR (median foci per cell pre-MR = 0.11, post-MR = 0.11, p = .90), but the number of 53BP1 foci significantly increased following MR (median foci per cell pre-MR = 0.46, post-MR = 0.54, p = .0140). Clinical and MR characteristics did not differ significantly between patients who had at least a 10% increase in foci per cell and those who did not. We conclude that MR exposure leads to a small (median 25%) increase in 53BP1 foci, however the clinical relevance of this increase is unknown and may be attributable to normal variation instead of MR exposure.

## Introduction

Magnetic resonance (MR) imaging has revolutionized medicine in general and, more recently, cardiovascular imaging in particular [1]. These advances in imaging appeared to have been achieved with minimal or no risk to the patient, in contrast to the known risks associated with ionizing radiation present with X-ray, Computed Tomography, and conventional angiographic modalities. However, multiple recent studies have raised an unexpected concern regarding MR safety because of potential damage to DNA [2–7]. Several of these studies quantified DNA damage in circulating human lymphocytes and other peripheral blood mononuclear cells (PBMCs) *in vivo* or *in vitro* following cardiac MR exams, and reported significant increases in the double-strand (ds) DNA damage marker γH2AX [3, 4]. While these studies suggested that DNA damage might be occurring in circulating PBMCs during cardiac MR, concern was expressed because the studies were small, only a single marker was examined, and downstream consequences were not determined [7].

Phosphorylation of the histone variant H2AX on Ser^139^, resulting in γH2AX, is one of the earliest cellular responses to ds DNA damage [8, 9]. γH2AX quantification has been widely applied to measure DNA damage from numerous radiological examination types known to impact DNA integrity [10]. Even though such DNA breaks are quickly repaired, it remains possible that imperfect repairs might lead to mutations associated with carcinogenesis [11–13].

Changes in downstream DNA repair process proteins can serve as additional markers for examining the effects of MR exams on lymphocyte DNA. Parallel to phosphorylation of H2AX, DNA damage mediators recruit repair proteins (Mre11/Rad50/Nbs1 and multiple myeloma SET domain proteins) that methylate histones. The DNA repair protein 53BP1 is then recruited to these methylated histones [14, 15], where it participates in DNA ds break repair by facilitating nonhomologous end-joining (the predominant DNA ds break repair mechanism in G0/G1 phase cells) and impairing BRCA1 function to inhibit homologous recombination repair (the predominant DNA ds break repair mechanism in S and G2 phase cells). DNA Studies that only measure changes in γH2AX may not be getting an accurate assessment of DNA damage and activation of repair, as DNA damage may be masked by robust 53BP1 activity [16]. Moreover, some DNA ds break repair has been shown to involve activation of 53BP1 independent of H2AX phosphorylation [9]. Because the formation of H2AX foci (signifying activation of ATM, ATR, or DNA-PK by various types of DNA damage, including DNA ds breaks) and 53BP1 foci (signifying both H2AX-dependent and–independent repair processes) assess two different aspects of DNA damage and repair [9], we reasoned that additional information might be gained by examining both proteins after MR exams.

Conflicting study results have prompted numerous investigators to call for carefully designed and reproducible experiments to discern the potential impact of cardiac MR scanning on DNA integrity [17]. Since there is a high degree of heterogeneity in γH2AX assay methodology, which may be a factor in the variation in study results [17], we elected to use an automated assay with a standardized method [18, 19]. The purpose of our study was to examine PBMCs from patients undergoing cardiac MR exams using automated quantification of the ds DNA damage and repair markers γH2AX and 53BP1 to further clarify the presence and extent of DNA damage associated with MR scanning.

## Materials and methods

Study design and implementation of this retrospective study were overseen by the Mayo Clinic institutional review board, conformed to Health Insurance Portability and Accountability Act guidelines on patient data integrity, and were carried out in accordance with the Declaration of Helsinki. Informed consent forms were obtained from all patients.

### Study design and population

This paired, prospective study involved obtaining paired blood samples from patients immediately before and after a clinically-indicated outpatient cardiac MR exam at our institution from April, 2014 to July, 2016. Outpatients were included if they 1) were scheduled to receive an outpatient gadolinium-enhanced cardiac MR exam for any indication and 2) could provide blood samples before and after the MR exam. Patients were excluded if they 1) were hospitalized at the time of MR exam, 2) had any history of malignancy, 3) were receiving chemotherapy or radiation therapy at the time of MR exam, 4) were taking steroids or other immunosuppressant drugs at the time of MR exam, or 5) could not provide consent.

### MRI exam

MRI exams were performed using a 1.5T GE Optima 450w scanner (GE Healthcare, Waukesha, WI) with a maximum gradient amplitude of 34mT/m and slew rate of 150 mT/m/ms. Patients underwent different imaging sequences depending on indication and radiologist preference. All patients underwent a gadolinium-enhanced MRI exam and received intravenous injections of Gadavist (Bayer AG, Leverkusen, Germany), Omniscan (GE Healthcare, Little Chalfont, UK), MultiHance (Bracco Imaging, Milan, Italy), Magnivist (Bayer AG), or Ablavar (Lantheus Medical Imaging, North Bilerica, MA). Detailed exam information, including individual sequences performed, time of sequence, and specific absorption rate (SAR), were collected for each patient. Specific absorptions (SA) were calculated for each sequence by multiplying sequence time (seconds) by SAR. Maximum SA, total SA, and highest SAR were calculated for each patient.

### Blood collection and analysis

Whole blood samples were obtained from each patient 30 minutes before the MR exam and within 30 minutes following the exam. A total of 3 ml of whole blood was obtained at each collection. Samples were placed on ice immediately following collection to halt any progression of DNA repair. Ficoll/hypaque gradient separation was subsequently performed to isolate peripheral blood mononuclear cells (PBMCs). In brief, samples were diluted in RPMI 1640 media (Corning Cellgro, Corning, NY) and Histopaque 1077 (Sigma-Aldrich, St. Louis, MO) was added to underlay the blood. The sample was centrifuged at 1200 rpm for 40 minutes at 18°C. Following removal of the buffy coat and a second centrifugation, the PBMCs were resuspended in 4% paraformaldehyde (Electron Microscopy Sciences, Hatfield, PA) and fixed on ice for 15 minutes. Following a third centrifugation, the samples were resuspended in phosphate-buffered saline (PBS, Thermo Fisher Scientific, Waltham, MA) and stored at 4°C until shipment to Exogen Biotechnology Inc. laboratory (Exogen). All samples were blinded prior to shipment and shipped on ice.

At Exogen, PBMCs were aliquoted into 96 well plates coated with proprietary reagent to allow adherence of fixed lymphocytes. Cells were then processed for immunostaining via programmed liquid handler (MultiFlo FX, BioTek, Winooksi, VT) as previously described [18, 19]. Briefly, cells were fixed with 2% paraformaldehyde in PBS for 5 min at room temperature followed by extensive washing (three washes with PBS for 5 min each). Cells were permeabilized with 1% Triton X-100 (Sigma-Aldrich) in PBS and blocked for 1 hr. with 3% BSA in PBS (Thermo Fisher Scientific) at room temperature. Cells were then incubated at room temperature with primary antibodies (1.33 μg/ml mouse monoclonal anti phospho-histone H2AX (Ser139) antibody (clone JBW301; Upstate Cell Signaling Solutions Inc. Charlottesville, VA) or 1:300 dilution rabbit polyclonal anti-53BP1 (IHC-00001, Bethyl Laboratories, Montgomery, TX) for 1 hr., and subsequently incubated with 5μg/mL secondary antibodies Alexa 488 anti-mouse (Thermo Fisher) or Alexa 488 anti-rabbit (Thermo Fisher). Nuclei were stained with 5μg/mL DAPI (Molecular Probes, Eugene, OR). Cells were washed with PBS for 5 minutes two times between all incubations. Cells were subjected to high throughput automated imaging and quantification using proprietary microscope equipment. Approximately 1000 cells per well were counted in technical duplicates per sample. Immunofluorescence data was consolidated and analyzed using Exogen’s automated foci quantification algorithm as previously described [19]. A wavelet morphological filter was applied to enhance foci peaks while reducing nonspecific signal noise. Nuclear space was identified by applying a constant threshold on the wavelet-filtered image and foci were identified using a background subtraction method. Touching foci were separated using a watershed algorithm [19].

### Statistical analysis

All statistical analyses were performed using JMP (version 10, SAS Institute, Cary, NC). Based on data from prior publications, we assumed the true change in γH2AX foci per cell (pre- vs. post-exam) is 0.001, with a standard deviation of 0.025 [2]. To test the hypothesis that there is no clinically significant change in γH2AX following cardiac MR exam, using a limit for the margin of inferiority set at 10% increase from baseline (0.0123), we had 90% power to reject the null hypothesis that a significant difference exists using a one-sided t-test (alpha = 0.025) with a total of 56 paired samples. We enrolled 60 patients to ensure our study was sufficiently powered. Changes in the number of pre- and post-MR γH2AX and 53BP1 foci within patient PMBCs were assessed using paired t-tests. Correlation within patients of relative changes in γH2AX versus 53BP1 was assessed using Pearson’s correlation coefficient. Differences between patients with >10% increase in γH2AX or 53BP1 foci within PBMCs and patients without this increase was assessed using the Wilcoxon rank-sum test or Pearson’s chi-squared test. Significance was assigned to differences of p < .05.

## Results

### Patient population

A total of 60 patients, 39 males (median age 54 (IQR 44–66)) and 21 females (median age 59 (36–69)) were enrolled in our study (Table 1). Patients predominantly underwent cardiac MR for cardiomyopathy or other cardiac indications, including coronary artery disease, tachycardia, and congenital cardiac disorders. Gadavist and Omniscan were most frequently used, with a median dose of 26 ml. Median MR exam time was 44 minutes (IQR: 34–58 min), median highest SAR was 1.48 W/kg (IQR: 1.35–1.61), median maximum specific absorption was 177 J/kg (IQR: 137–259), and median total specific absorption was 472 J/kg (IQR: 352–565).

**Table 1:** Study population.

**Patient**	
**N**	60
**Age, years: median (IQR)**	55 (44–68)
Male patients	54 (44–66)
Female patients	59 (36–69)
**Number of Female patients (%)**	21 (35)
**Cardiac MR indication (%)**	
Hypertrophic cardiomyopathy	9 (15)
Other cardiomyopathy	9 (15)
History of Afib	8 (13)
Pericarditis	6 (10)
Other*	28 (47)
**MR exam**	
**Gadolinium agent (%)**	
Gadavist	24 (40)
Omniscan	22 (37)
MultiHance	12 (20)
Ablavar	1 (1.7)
Magnavist	1 (1.7)
**Gadolinium dose, ml: median (IQR)**	26 (18–36)
**Total MR exam time, min: median (IQR)**	44 (34–58)
**Highest SAR, W/kg: median (IQR)**	1.48 (1.35–1.61)
**Maximum specific absorption; Time X SAR, J/kg: median (IQR)**	177 (137–259)
**Total specific absorption; Time X SAR, J/kg: median (IQR)**	472 (352–565)

*”Other” indications included coronary artery disease, tachycardia, and congenital cardiac disorders.

### Effect of cardiac MR exams on changes in H2AX and 53BP1

**Fig 1:**
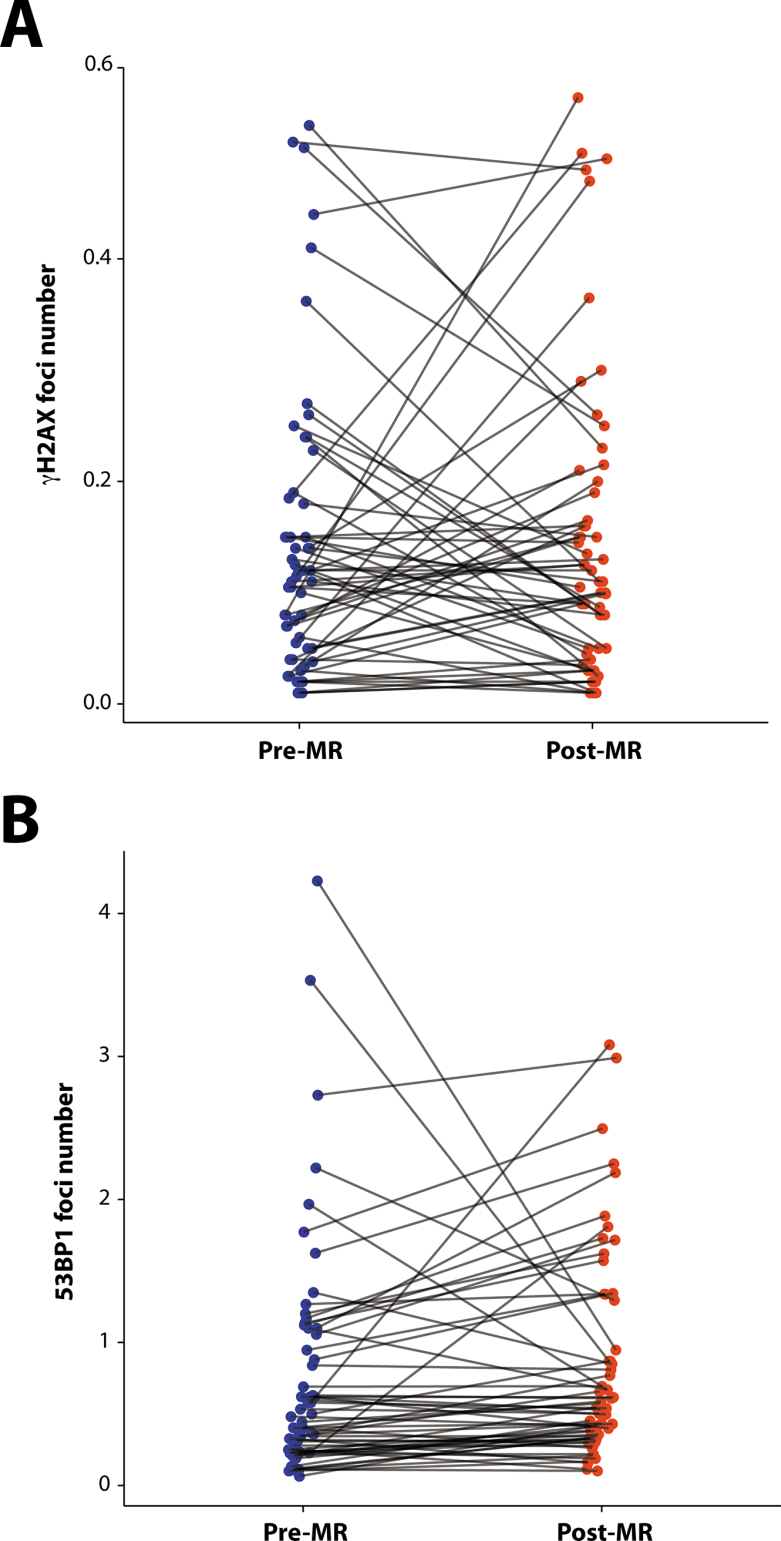
**Individual changes in γH2AX (A) and 53BP1 (B) foci per cell following cardiac MR**.

**Fig 2:**
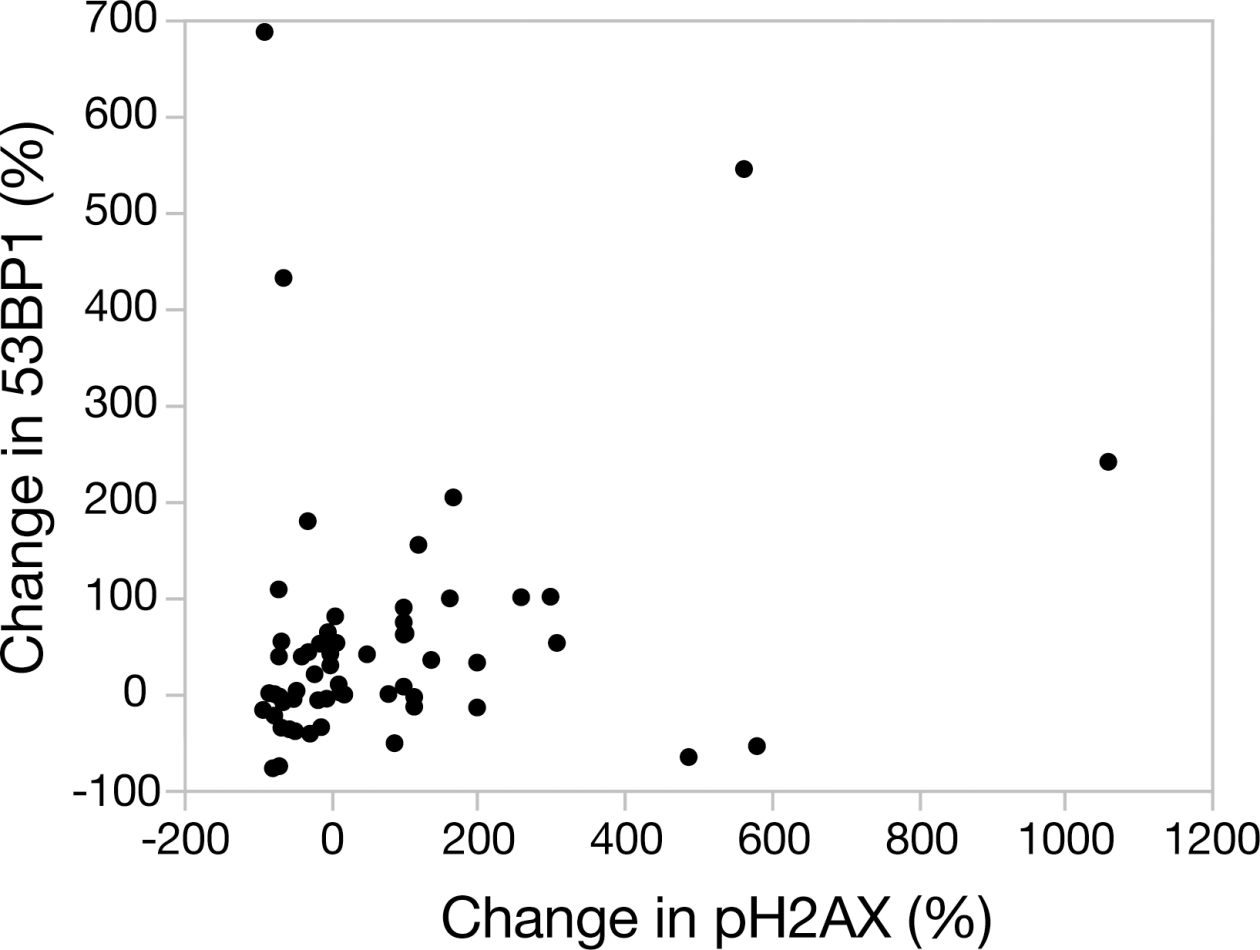
Correlation between relative changes in γH2AX and 53BP1 within patients.

Examples of γH2AX and 53BP1 staining of PBMCs are shown in S1 Fig. The change in PBMC γH2AX and 53BP1 foci per cell following MR exam in individual patients is shown in Fig 1. The number of γH2AX foci in patient samples did not significantly change following cardiac MR (Table 2, median foci per cell pre-MR = 0.11, post-MR = 0.11, p = .90). However, the number of 53BP1 foci significantly increased following MR (median foci per cell pre-MR = 0.46, post-MR = 0.54, p = 0.0140). A total of 40% (23/58) and 28% (16/58) of patients had at least a 50% increase or decrease, respectively, in γH2AX following MR, and a total of 35% (21/60) and 8.3% (5/60) of patients had at least a 50% increase or decrease, respectively, in 53BP1 following MR. No significant correlation between relative changes in γH2AX foci and 53BP1 foci within patients were found (Fig 2, ρ = 0.27, p = .09).

**Table 2:** Changes in H2AX and 53BP1 foci per cell following cardiac MR exam.

	n	Pre-MR	Post-MR	Absolute change	Relative change	P value*
**γH2AX foci/cell number (median, IQR)**	58	0.11 (0.04–0.17)	0.11 (0.04–0.19)	0 (-0.09–0.07)	0 (-58%-114%)	0.90
**53BP1 foci/cell number (median, IQR)**	60	0.46 (0.24–1.09)	0.54 (0.33–1.21)	0.07 (-0.04–0.35)	25% (-8.4%-63%)	**.0140**

*Calculated using Wilcoxon signed rank test.

Patients who had at least a 10% increase in γH2AX (n = 26) and 53BP1 (n = 31) foci after their MR exam were compared to patients without this increase (Tables 3 and 4). No patient characteristic or MR procedural characteristic was found to correlate with increases in γH2AX or 53BP1 foci.

**Table 3:** Characteristics of patients with >10% increase in γH2AX.

Patient	γH2AX increase	No γH2AX increase	P value
N*	26	32	
**Age, years: median (IQR)**	53 (42–63)	58 (44–69)	.40
**Number of Females (%)**	8 (31)	13 (41)	.44
**Cardiac MR indication (%)**			.67
Cardiomyopathy	8 (31)	9 (28)	
History of Afib	5 (19)	3 (9.4)	
Pericarditis	2 (7.7)	4 (13)	
Other**	11 (42)	16 (50)	
**MR exam**			
**Gadolinium agent (%)**			.39
Gadavist	8 (31)	16 (50)	
Omniscan	11 (42)	11 (34)	
MultiHance	5 (19)	5 (16)	
Ablavar	1 (3.9)	0	
Magnavist	1 (3.9)	0	
**Gadolinium dose, ml: median (IQR)**	26 (19–36)	24 (18–36)	.86
**Highest SAR, W/kg: median (IQR)**	1.45 (1.29–1.66)	1.49 (1.40–1.61)	.58
**Maximum specific absorption; Time X SAR, J/kg: median (IQR)**	170 (138–274)	190 (141–259)	.90
**Total specific absorption; Time X SAR, J/kg: median (IQR)**	477 (346–619)	475 (355–555)	.93

*n = 58, 2 patients did not have post-MR γH2AX results.

**”Other” indications included coronary artery disease, tachycardia, and congenital cardiac disorders.

**Table 4:** Characteristics of patients with >10% increase in 53BP1.

Patient	53BP1 increase	No 53BP1 increase	P value
**N**	31	29	
**Age, years: median (IQR)**	59 (45–69)	53 (40–66)	.32
**Number of Females (%)**	9 (29%)	12 (41%)	.32
**Cardiac MR indication (%)**			.72
Cardiomyopathy	8 (26)	10 (34)	
History of Afib	5 (16)	3 (10)	
Pericarditis	4 (13)	2 (6.9)	
Other*	14 (45)	14 (48)	
**MR exam**			
**Gadolinium agent (%)**			.62
Gadavist	12 (39)	12 (41)	
Omniscan	13 (42)	9 (31)	
MultiHance	6 (19)	6 (21)	
Ablavar	0	1 (3.5)	
Magnavist	0	1 (3.5)	
**Gadolinium dose, ml: median (IQR)**	28 (18–36)	24 (18–36)	.62
**Highest SAR, W/kg: median (IQR)**	1.48 (1.36–1.62)	1.48 (1.33–1.60)	.96
**Maximum specific absorption; Time X SAR, J/kg: median (IQR)**	166 (122–257)	179 (142–271)	.29
**Total specific absorption; Time X SAR, J/kg: median (IQR)**	490 (324–619)	496 (355–538)	.35

*”Other” indications included coronary artery disease, tachycardia, and congenital disorders

## Discussion

Our single center, prospective study demonstrates that clinically ordered gadolinium-enhanced cardiac MR exams were not associated with a significant increase in the DNA damage marker γH2AX in patient blood samples. A significant increase in foci of the DNA repair protein 53BP1 was observed post-MR, however this increase was small and there was no correlation between patients with an increase in 53BP1 and patients with an increase in γH2AX. There was also no correlation between patients with an increase in γH2AX or 53BP1 and patient or MR exam characteristics including age, gender, indication, gadolinium agent or dose, specific absorption rate, or specific absorption. **Collectively, these findings raise the possibility that gadolinium-enhanced cardiac MR exams may activate DNA repair, however the observed 53BP1 changes may instead be attributable to normal variance.** Examining only DNA damage markers, such as γH2AX, may give incomplete information concerning activation of the DNA damage response after cardiac MR.

There is much conflicting evidence regarding the effect of MR exposure on ds DNA damage in lymphocytes. Simi et al. found an immediate increase in DNA damage, based on increased numbers of micronuclei, after cardiac MR followed by a decrease at 48 hours [6] and Lee et al. found increased numbers of micronuclei correlated with the length of MR [20], while Szerencsi et al. found no damage using a comet assay and measuring micronuclei [21]. Yildiz found a significant increase in DNA damage in lymphocytes based on an alkaline comet assay [22]. Measuring in lymphocytes, Fietcher et al. found an immediate increase in γH2AX foci per cell in lymphocytes following cardiac MR [3], while Lancellotti et al. found no increase in γH2AX foci in PBMCs after an hour but an increase after both two days and one month [4]. Five studies, however, found no increase in γH2AX foci in lymphocytes in post-MR timeframes ranging from five minutes to 72 hours using MR scanner strengths ranging from 3T to 7T [2, 5, 23–25]. Whether the study was positive or negative for ds DNA damage was not dependent on MR field strength, use of contrast, or whether the experiment was *in vivo* or *in vitro*. Time between MR exposure and sampling was also inconsistently related to significant increases in γH2AX. This suggests that either experimental conditions among these studies were too heterogeneous to adequately compare results or that γH2AX increases are inconsistently seen following MR exposure.

To our knowledge, our study is the first to examine the effects of MR exposure on DNA repair markers such as 53BP1. Our finding of a significant increase in 53BP1 foci per cell but not in γH2AX foci per cell following cardiac MR exams raises the possibility that MR exposure may cause DNA damage, however several of our findings argue against this hypothesis. First, the median increase in number of foci was only 25% and remained below three 53BP1 foci per cell in the majority of cases (Fig 1). This observation, coupled with the fact that DNA repair laboratories often consider nuclei negative for foci unless 8–10 foci per cell are visible, raises questions about the biological importance of the 53BP1 foci. While they might reflect subtle activation of DNA repair, it is also possible that the 53BP1 increase we observed could be attributable to normal variability. Second, we were unable to find any relationship between patients with an increase in 53BP1 foci number and patient and MR procedural characteristics. Cumulatively, our findings must be interpreted cautiously and not as definitive evidence that MR exposure results in DNA damage. Additional studies of MR exposure that include 53BP1 and other DNA damage and repair markers are necessary to understand the biological importance of these observations.

Our study improves upon prior MR DNA damage studies in several other ways. First, to our knowledge our study is the first to use an automated assay to quantify γH2AX and 53BP1 foci per cell. Manual immunofluorescence analysis is extremely tedious, time consuming, and operator dependent, prompting experts to call for a standardization of γH2AX assessment [12]. Second, our study is to our knowledge the largest study of the effects of MR exposure on DNA damage *in vivo* to date and is sufficiently powered to confirm a lack of change in γH2AX following MR. Third, we obtained post-MR blood samples within 30 minutes following completion of the MR exam, which provided sufficient time for a potential DNA damage and repair response to be activated and detected. γH2AX foci generally increase several minutes after lesion creation, reach a maximum after 30 minutes, and fall to previous levels within 24 hours [17].

Our study had several limitations. First, while our study was sufficiently powered to detect no significant difference in γH2AX in our cohort, our sample size was small for comparing clinical and MR procedural characteristics between patients with an increase in γH2AX or 53BP1 and patients without an increase. Second, we only examined patients who underwent a gadolinium-enhanced MR exam; we therefore could not compare γH2AX and 53BP1 changes between patients who received gadolinium-enhanced MR exams and patients who received unenhanced MR exams. Third, our cohort underwent different MR sequences, specific absorption, and specific absorption rates, and received several different types of gadolinium. Fourth, we did not examine changes in γH2AX or 53BP1 in control patients not exposed to MR, which would help determine whether the significant increase in 53BP1 foci following MR is truly attributable to MR. Finally, we did not examine changes in γH2AX or 53BP1 at later time points to determine whether and when 53BP1 levels return to pre-MR baseline levels. Additional studies are necessary to validate our findings.

## Conclusions

Our prospective study of patients undergoing gadolinium-enhanced cardiac MR exams found no significant changes in the number of foci of the ds DNA damage marker γH2AX but a significant, albeit small (median 25%), increase in foci formed by the DNA repair protein 53BP1. This increase is of questionable clinical relevance and may be attributable to normal variance in 53BP1. Additional studies are needed to validate these findings, examine changes in γH2AX and 53BP1 over a longer post-MR timeframe, assess changes in other DNA repair proteins, and examine changes in these markers in control patients not subjected to MR exposure. Because DNA damage and repair can occur independently of H2AX phosphorylation, future studies should examine both DNA damage and repair markers to get a more complete picture of the effects of MR exposure on DNA.

## Supporting information

S1 Fig Representative peripheral blood mononuclear cell staining of 53BP1 and γH2AX. Nuclei stained blue by DAPI are shown. White foci within the nuclei are 53BP1 or γH2AX foci as visualized by anti-53BP1 or anti-γH2AX antibodies as described in the Methods. These foci were detected by Exogen’s automated foci quantification algorithm as highlighted in red.
(TIF)

## References

[1] Greulich S, Arai AE, Sechtem U, Mahrholdt H. Recent advances in cardiac magnetic resonance. F1000Res. 2016;5 doi: 10.12688/f1000research.8383.1 27635240 10.12688/f1000research.8383.1 PMC5017285

[2] Brand M, Ellmann S, Sommer M, May MS, Eller A, Wuest W, et al Influence of cardiac MR imaging on DNA double-strand breaks in human blood lymphocytes. Radiology. 2015;277(2):406–412. doi: 10.1148/radiol.2015150555 26225451 10.1148/radiol.2015150555

[3] Fiechter M, Stehli J, Fuchs TA, Dougoud S, Gaemperli O, Kaufmann PA. Impact of cardiac magnetic resonance imaging on human lymphocyte DNA integrity. Eur Heart J. 2013;34(30):2340–2345. doi: 10.1093/eurheartj/eht184 23793096 10.1093/eurheartj/eht184 PMC3736059

[4] Lancellotti P, Nchimi A, Delierneux C, Hego A, Gosset C, Gothot A, et al Biological effects of cardiac magnetic resonance on human blood cells. Circ Cardiovasc Imaging. 2015;8(9):e003697 doi: 10.1161/CIRCIMAGING.115.003697 26338876 10.1161/CIRCIMAGING.115.003697

[5] Reddig A, Fatahi M, Friebe B, Guttek K, Hartig R, Godenschweger F, et al Analysis of DNA double-strand breaks and cytotoxicity after 7 tesla magnetic resonance imaging of isolated human lymphocytes. PLoS One. 2015;10(7):e0132702 doi: 10.1371/journal.pone.0132702 26176601 10.1371/journal.pone.0132702 PMC4503586

[6] Simi S, Ballardin M, Casella M, De Marchi D, Hartwig V, Giovannetti G, et al Is the genotoxic effect of magnetic resonance negligible? Low persistence of micronucleus frequency in lymphocytes of individuals after cardiac scan. Mutat Res. 2008;645(1–2):39–43. doi: 10.1016/j.mrfmmm.2008.08.011 18804118 10.1016/j.mrfmmm.2008.08.011

[7] Vijayalaxmi, Fatahi M, Speck O. Magnetic resonance imaging (MRI): A review of genetic damage investigations. Mutat Res Rev Mutat Res. 2015;764:51–63. doi: 10.1016/j.mrrev.2015.02.002 26041266 10.1016/j.mrrev.2015.02.002

[8] Scully R, Xie A. Double strand break repair functions of histone H2AX. Mutat Res. 2013;750(1–2):5–14. doi: 10.1016/j.mrfmmm.2013.07.007 23916969 10.1016/j.mrfmmm.2013.07.007 PMC3818383

[9] Yuan J, Adamski R, Chen J. Focus on histone variant H2AX: to be or not to be. FEBS Lett. 2010;584(17):3717–3724. doi: 10.1016/j.febslet.2010.05.021 20493860 10.1016/j.febslet.2010.05.021 PMC3695482

[10] Kuefner MA, Brand M, Engert C, Schwab SA, Uder M. Radiation induced DNA double-strand breaks in radiology. Rofo. 2015;187(10):872–878. doi: 10.1055/s-0035-1553209 26333102 10.1055/s-0035-1553209

[11] Hill MA, O'Neill P, McKenna WG. Comments on potential health effects of MRI-induced DNA lesions: quality is more important to consider than quantity. Eur Heart J Cardiovasc Imaging. 2016;17(11):1230–1238. doi: 10.1093/ehjci/jew163 27550664 10.1093/ehjci/jew163 PMC5081138

[12] Valdiglesias V, Giunta S, Fenech M, Neri M, Bonassi S. gammaH2AX as a marker of DNA double strand breaks and genomic instability in human population studies. Mutat Res. 2013;753(1):24–40. doi: 10.1016/j.mrrev.2013.02.001 23416207 10.1016/j.mrrev.2013.02.001

[13] Yu T, MacPhail SH, Banath JP, Klokov D, Olive PL. Endogenous expression of phosphorylated histone H2AX in tumors in relation to DNA double-strand breaks and genomic instability. DNA Repair (Amst). 2006;5(8):935–946. doi: 10.1016/j.dnarep.2006.05.040 16814620 10.1016/j.dnarep.2006.05.040

[14] Rasche L, Heiserich L, Behrens JR, Lenz K, Pfuhl C, Wakonig K, et al Analysis of lymphocytic DNA damage in early multiple sclerosis by automated gamma-H2AX and 53BP1 foci detection: A case control study. PLoS One. 2016;11(1):e0147968 doi: 10.1371/journal.pone.0147968 26820970 10.1371/journal.pone.0147968 PMC4731473

[15] Watts FZ. Repair of DNA double-strand breaks in heterochromatin. Biomolecules. 2016;6(4):47 doi: 10.3390/biom6040047 27999260 10.3390/biom6040047 PMC5197957

[16] Adams MM, Carpenter PB. Tying the loose ends together in DNA double strand break repair with 53BP1. Cell Div. 2006;1:19 doi: 10.1186/1747-1028-1-19 16945145 10.1186/1747-1028-1-19 PMC1601952

[17] Sanchez-Flores M, Pasaro E, Bonassi S, Laffon B, Valdiglesias V. gammaH2AX assay as DNA damage biomarker for human population studies: defining experimental conditions. Toxicol Sci. 2015;144(2):406–413. doi: 10.1093/toxsci/kfv011 25616596 10.1093/toxsci/kfv011

[18] Costes SV, Boissiere A, Ravani S, Romano R, Parvin B, Barcellos-Hoff MH. Imaging features that discriminate between foci induced by high- and low-LET radiation in human fibroblasts. Radiat Res. 2006;165(5):505–515. doi: 10.1667/RR3538.1 16669704 10.1667/RR3538.1

[19] Neumaier T, Swenson J, Pham C, Polyzos A, Lo AT, Yang P, et al Evidence for formation of DNA repair centers and dose-response nonlinearity in human cells. Proc Natl Acad Sci U S A. 2012;109(2):443–448. doi: 10.1073/pnas.1117849108 22184222 10.1073/pnas.1117849108 PMC3258602

[20] Lee JW, Kim MS, Kim YJ, Choi YJ, Lee Y, Chung HW. Genotoxic effects of 3 T magnetic resonance imaging in cultured human lymphocytes. Bioelectromagnetics. 2011;32(7):535–542. doi: 10.1002/bem.20664 21412810 10.1002/bem.20664

[21] Szerencsi A, Kubinyi G, Valiczko E, Juhasz P, Rudas G, Mester A, et al DNA integrity of human leukocytes after magnetic resonance imaging. Int J Radiat Biol. 2013;89(10):870–876. doi: 10.3109/09553002.2013.804962 23679232 10.3109/09553002.2013.804962

[22] Yildiz S, Cece H, Kaya I, Celik H, Taskin A, Aksoy N, et al Impact of contrast enhanced MRI on lymphocyte DNA damage and serum visfatin level. Clin Biochem. 2011;44(12):975–979. doi: 10.1016/j.clinbiochem.2011.05.005 21620817 10.1016/j.clinbiochem.2011.05.005

[23] Fatahi M, Reddig A, Vijayalaxmi, Friebe B, Hartig R, Prihoda TJ, et al DNA double-strand breaks and micronuclei in human blood lymphocytes after repeated whole body exposures to 7T Magnetic Resonance Imaging. Neuroimage. 2016;133:288–293. doi: 10.1016/j.neuroimage.2016.03.023 26994830 10.1016/j.neuroimage.2016.03.023

[24] Reddig A, Fatahi M, Roggenbuck D, Ricke J, Reinhold D, Speck O, et al Impact of in vivo high-field-strength and ultra-high-field-strength MR imaging on DNA double-strand-break formation in human lymphocytes. Radiology. 2017;282(3):782–789. doi: 10.1148/radiol.2016160794 27689924 10.1148/radiol.2016160794

[25] Schwenzer NF, Bantleon R, Maurer B, Kehlbach R, Schraml C, Claussen CD, et al Detection of DNA double-strand breaks using gammaH2AX after MRI exposure at 3 Tesla: an in vitro study. J Magn Reson Imaging. 2007;26(5):1308–1314. doi: 10.1002/jmri.21138 17969164 10.1002/jmri.21138

