# Correction: Gadolinium-enhanced cardiac MR exams of human subjects are associated with significant increases in the DNA repair marker 53BP1, but not the damage marker γH2AX

**DOI:** 10.1371/journal.pone.0193634

**Published:** 2018-02-23

**Authors:** Jennifer S. McDonald, Robert J. McDonald, Jacob B. Ekins, Antony S. Tin, Sylvain Costes, Tamara M. Hudson, Dana J. Schroeder, Kevin Kallmes, Scott H. Kaufmann, Philip M. Young, Aiming Lu, Ramanathan Kadirvel, David F. Kallmes

The fourth author’s name is spelled incorrectly in the byline and Funding section. The correct name is: Antony S. Tin.

## References

[1] McDonald JS, McDonald RJ, Ekins JB, Tin AS, Costes S, Hudson TM, et al (2018) Gadolinium-enhanced cardiac MR exams of human subjects are associated with significant increases in the DNA repair marker 53BP1, but not the damage marker γH2AX. PLoS ONE 13(1): e0190890 https://doi.org/10.1371/journal.pone.0190890 29309426 10.1371/journal.pone.0190890 PMC5757995

