# Supplementary figures and images for: Gadolinium-enhanced cardiac MR exams of human subjects are associated with significant increases in the DNA repair marker 53BP1, but not the damage marker γH2AX
Source: PLoS One. 2018 Jan 8;13(1):e0190890. doi: 10.1371/journal.pone.0190890 (PMC5757995; doi:10.1371/journal.pone.0190890)

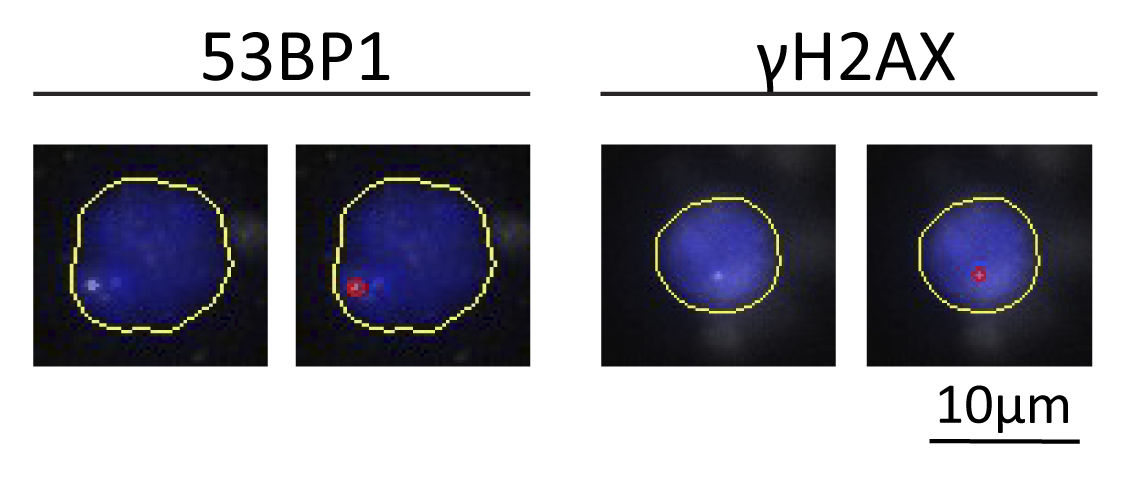

Supplement: S1 Fig — Nuclei stained blue by DAPI are shown. White foci within the nuclei are 53BP1 or γH2AX foci as visualized by anti-53BP1 or anti-γH2AX antibodies as described in the Methods. These foci were detected by Exogen’s automated foci quantification algorithm as highlighted in red. (TIF) [file pone.0190890.s001.tif]
